# Reconciliation and Indigenous self-determination in health research: A call to action

**DOI:** 10.1371/journal.pgph.0000999

**Published:** 2022-09-01

**Authors:** Pamela Roach, Faye McMillan

**Affiliations:** 1 Department of Family Medicine, Cumming School of Medicine, University of Calgary, Calgary, Canada; 2 Department of Community Health Sciences, Cumming School of Medicine, University of Calgary, Calgary, Canada; 3 Métis Nation of Alberta; 4 School of Population Health, Faculty of Medicine and Health, University of New South Wales, Kensington, Australia; 5 Office of the National Rural Health, Department of Health and Aging, Cairns, Australia; 6 Wiradjuri Nation of Australia; PLOS: Public Library of Science, UNITED STATES

As Section Editors of the Indigenous Health section of *PLOS Global Public Health*, we were asked to think about the urgent health priorities facing Indigenous people around the world. A challenging task as the diversity amongst Indigenous people is great; and local relationships with structures, policy, our relatives, and the land will create greater diversity in the health challenges we face. A common challenge for most, if not all, Indigenous people globally is the ongoing impact of colonialism and systemic racism encountered within our health care systems.

There is irrefutable evidence that Indigenous people living in Canada and Australia experience racism in the healthcare system [1–4]. In 2015, the Truth and Reconciliation Commission of Canada (TRC) released 94 Calls to Action compelling Canadian institutions across service sectors (i.e., education, child welfare, criminal, justice, healthcare) to acknowledge that colonization is the principal driver of population-level health disparities experienced by Indigenous people [5].

Within Australia, the Mayi Kuwayu Longitudinal Study is a ground-breaking study that is examining the centrality of culture and is asking what culture means for Aboriginal and Torres Strait Islander Peoples. Data collected by the Mayi Kuwayu study between 2018–2020 highlighted the breadth and the extent of racism on the health and wellbeing of Aboriginal and Torres Strait Islander people, and that across the board links with racism were identified within mental health, physical health and cultural wellbeing [6]. Additionally, evidence shows that Indigenous self-determination is an important factor in maintaining well-being and contributes to positive health outcomes [7]. These rights to self-determination and sovereignty are further entrenched to the UN Declaration of Rights for Indigenous People [8] and defined there as:

*Article 3 Indigenous peoples have the right to self-determination. By virtue of that right they freely determine their political status and freely pursue their economic, social and cultural development*.

*Article 4 Indigenous peoples, in exercising their right to self-determination, have the right to autonomy or self-government in matters relating to 4.Resolution 217 A (III). 5 their internal and local affairs, as well as ways and means for financing their autonomous functions*.

From a research and publishing perspective there is an urgent need to call out ‘black wallpapering’ and tokenism of research methodologies and publications that make them appear to be culturally inclusive and responsive than they really are. As pointed out in a recent Opinion article in *PLOS Global Public Health* [9], Indigenous voices and perspectives are often missing in global health, even when the focus is on ‘decolonizing’ global health. So then we asked ourselves, what is our role as editorial board members and academics working in Indigenous health? What can we do to challenge the erasure of Indigenous people?

We know that global health research and public health research more broadly have a history of doing research ‘for’ or ‘on’ people rather than ‘with’ people [10]. We see this globally with Indigenous populations, who are said to be marginalized, vulnerable and underrepresented on their own homelands through centuries of colonization and assimilation. This has embedded colonialism and epistemic racism throughout all of our systems, including healthcare, health policy and health research. This manifests in the way Indigenous health research is designed, undertaken and disseminated; including in peer reviewed journals. In order to authentically work toward decolonizing global health and Indigenous health we must respect the sovereignty of Indigenous peoples in all contexts.

A growing number of journals, including PLOS journals, are discouraging parachute or helicopter research [11], or calling for author reflexivity statements [12] while other journals have initiated calls for transparent positionality as a required component for Indigenous health research [13]. We support these calls that have been presented in the *Australian Journal of Rural Health*, *Rural & Remote Health*, and the *Canadian Journal of Rural Medicine* [13] and we echo that call for Indigenous health globally.

To be clear: we call for journals that publish Indigenous health research to have indicators for each author to indicate the Indigenous members of the team or the Indigenous communities that have contributed to the work as a requirement for publication. If knowledge is held by the community, we should be making space as academic publishers for communities to contribute collectively as an author and for authors to list their community as a recognized affiliation, as stated by Victor Lopez-Carmen[14]:

*Let’s normalize being part of an Indigenous Nation as perfectly valid author affiliations in academia. Indigenous Peoples are their own experts and have expertise grounded in community and traditional knowledge*.

Further detail around contribution and connection to community needs to be provided in the author contribution and acknowledgment sections to avoid the tokenism that is often at play in academic publishing. Indigenous peoples can and do lead the work that is needed, and peer reviewed journals have a role to play in ensuring accountability from researchers and publishers alike. Cultural content, and indeed, cultural safety and sovereignty needs to be embedded into research and go beyond just identifying the Indigeneity of publication authors so that published Indigenous health research is expected to be Indigenous-led, co-designed and managed with the communities impacted by the research.

Though creating publishing requirements only addresses one part of the research enterprise, we believe that this will create the momentum that leads to truly ethical research relationships with Indigenous people by requiring ethical ways of working in research prior to the publication phase. We hope these conversations create space for all researchers, funding agencies and journals to explore options to broaden this position statement beyond the publishing stage so the research process is culturally robust from conception to completion to avoid ‘end of pipeline’ rhetoric and tokenism.

Specifically, Indigenous knowledges, ethics, processes and protocols need to be embedded into all stages of the research process [15], including:

○ cultivating community owned proposals;
○ research team recruitment and leadership;
○ culturally responsive methodologies;
○ strengthening community capacity;
○ delivering community focused outcomes;
○ communicating outcomes with cultural appropriate knowledge exchange medias; and
○ following up and engaging communities on research impact including impact evaluation approaches established at project outset.

These moves to equity also help efforts to diversify and decolonize global health teaching when identifying the positionality of authors–to embed this into open access journals with equitable fee structures elevates research led by diverse scholars around the world in a way that is accessible and resists current academic power structures. This is also an important gesture of active reconciliation given the history of research on Indigenous people and health without prior and informed consent, engagement and respect for culture, lore and Ways of Knowing. From this point, we also want to continue the dialogue to create a more holistic position statement co-developed under a forum that is Indigenous led with diverse representation from our equally diverse communities and research networks.

As Indigenous health Section Editors we believe in the principal of “nothing about us without us” with regards to Indigenous research. Accordingly, we welcome and want to support manuscripts that are Indigenous-led and describe authentic partnerships with Indigenous communities, individuals and researchers. We also support the inclusion of diverse Indigenous methodologies and worldviews to expand and support the contributions of Indigenous scholars around the world to the scientific community.
